# Supplementary material for: From Shunt to Recovery: A Multidisciplinary Approach to Hydrocephalus Treatment in Severe Acquired Brain Injury Rehabilitation
Source: Brain Sci. 2021 Dec 21;12(1):3. doi: 10.3390/brainsci12010003 (PMC8773713; doi:10.3390/brainsci12010003)
Supplement: Supplementary file 1 [file brainsci-12-00003-s001.zip › brainsci-1461460-supplementary.pdf]

Table S1. Clinical features of patients undergoing CSF shunt.

**Neurological worsening**
Decreases the length of time the patient maintains arousal
Exhibit sustained eyelid closure
Stops following commands
Behavioural responsiveness ceases despite sustained eye opening
Cognitive and motor slowdown
**Ceased clinical improvement**
Hypertonia increase
Deglutition worsening
Gastrointestinal symptoms (regurgitation, vomiting, slower gastric transit)

Table S2. Factors associated with VPS complications in the subgroup of patients treated with VPS during rehabilitation. Results from logistic regression analyses.

	VPS complications	
	OR (95%CI)	p-value
Time to diagnosis (weeks)§	0.91 (0.81 – 1.03)	0.124
Time to surgery (weeks)§	0.99 (0.81 – 1.20)	0.891

Note: Time to diagnosis indicates time between admission and hydrocephalus diagnosis; §one patient with timing >200 days was excluded.
